# A real-world test of artificial intelligence infiltration of a university examinations system: A “Turing Test” case study

**DOI:** 10.1371/journal.pone.0305354

**Published:** 2024-06-26

**Authors:** Peter Scarfe, Kelly Watcham, Alasdair Clarke, Etienne Roesch

**Affiliations:** 1 School Psychology and Clinical Language Sciences (PCLS), University of Reading, Reading, United Kingdom; 2 Department of Psychology, University of Essex, Colchester, United Kingdom; Ascension Sacred Heart Hospital Pensacola, UNITED STATES

## Abstract

The recent rise in artificial intelligence systems, such as ChatGPT, poses a fundamental problem for the educational sector. In universities and schools, many forms of assessment, such as coursework, are completed without invigilation. Therefore, students could hand in work as their own which is in fact completed by AI. Since the COVID pandemic, the sector has additionally accelerated its reliance on unsupervised ‘take home exams’. If students cheat using AI and this is undetected, the integrity of the way in which students are assessed is threatened. We report a rigorous, blind study in which we injected 100% AI written submissions into the examinations system in five undergraduate modules, across all years of study, for a BSc degree in Psychology at a reputable UK university. We found that 94% of our AI submissions were undetected. The grades awarded to our AI submissions were on average half a grade boundary higher than that achieved by real students. Across modules there was an 83.4% chance that the AI submissions on a module would outperform a random selection of the same number of real student submissions.

## Introduction

### What is artificial intelligence?

One of the key goals of the field of artificial intelligence (AI) is to create an artificial system that generates intelligent reasoning in natural language based upon ingested multi-modal external data. This goal is exemplified by the ‘imitation game’ published by Alan Turing, which went on to be known as the ‘Turing Test’. In its original form [1] a human interrogator has a text-based conversation with a man and a woman in another room. The interrogator knows these only as X and Y. The goal for the interrogator is to determine which of X and Y is a man and which a woman. Turing asked, what would happen if either X or Y was replaced by a machine? Will the interrogator decide incorrectly as often as when the game is played with two humans? Turing’s goal was to use this to determine whether machines can think i.e. a functional definition of what we now term AI.

Turing’s thought experiment in some ways established the field of AI, where systems are developed to try and emulate the process of human thought and reasoning. The ‘Turing Test’ is now more generally used to refer to our ability to be able to distinguish artificial from human intelligence. Early AI systems tried to achieve artificial reasoning by manipulating symbols representing real world concepts with a predefined set of rules. Examples of this approach included the ‘Logic Theory Machine’ [2] and the ‘General Problem Solver’ [3]. This manual programming approach failed in its goal of mimicking human intelligence, which could not easily be expressed in terms of a set of predefined concepts and symbolic rules. This led to scepticism about whether the ultimate goals of AI were at all possible [4, 5].

Symbolic AI systems became termed ‘Good Old Fashion AI’ [6] in contrast to alternative ‘artificial neural network’ (ANN) based approaches, which were originally developed in the 1950s and laid the foundation for today’s AI. ANNs consist of a set of nodes which are connected to one another, analogous to the way in which the brain consists of a set of neurons which are connected to one another as a network. ANNs have an input and produce an output, the output being determined by the connectivity between nodes and the connection strengths (the nodes themselves being far simpler than neurones). ANNs can be trained in various ways to update these weights using specific algorithms so as to produce the desired output e.g., supervised, unsupervised, or semi-supervised learning [7].

Early networks were “shallow” in that they contained only a few layers of nodes but were able to complete tasks such as binary classification, regression, and simple feature extraction. They could not however complete tasks akin to more complex human reasoning. This changed with the advent of “deep neural networks” (DNNs) during the 2010’s. DNN’s work on the same core principles as early ANN’s but contain more nodes and layers and have a more complicated computational architecture. This allows the network to extract more abstract features of the input and complete more complicated tasks. As of this writing, we are undergoing somewhat of an AI revolution, where DNNs are used for a variety of tasks such as, recognising objects in images [8], controlling autonomous vehicles [9], modelling protein folding [10], playing complex high dimensional games [11], language translation [12], speech to text conversion [13], and natural language understanding [14], just to name just a few.

### AI, education and academic misconduct

There has been an explosion of interest in AI over the last few years, driven largely by the emergence of more complex “large language models” (LLM’s), a type of AI designed to understand and generate human language. The most widely discussed of these, both in the academic literature and public discourse, is ChatGPT created by OpenAI (at the time of writing, the latest model being GPT-4). In GPT-4’s own words (in the response to the prompt “What is ChatGPT?”):

*“ChatGPT*, *or Chatbot Generative Pre-trained Transformer*, *is an advanced AI language model developed by OpenAI*. *It is based on the GPT architecture*, *with the latest version being GPT-4 at the time of your question*. *These models are designed to understand and generate human-like text*, *allowing them to engage in conversations*, *answer questions*, *and provide information on various topics*.

*ChatGPT is pre-trained on a large corpus of text from the internet*, *which enables it to have a broad understanding of language and knowledge about various topics up to a certain date (in this case*, *September 2021)*. *The model is fine-tuned with additional training data to make it more useful and safe in conversational contexts*.

*Despite its impressive capabilities*, *ChatGPT may still make mistakes or generate incorrect or outdated information*, *as it relies on the quality of the data it was trained on and does not possess an ability to learn new information or update itself in real-time*.*”*

LLMs such as GPT-4 are based upon the widely used transformer architecture [15] and exploit the simple fact that human language is non-random and follows a stable structure. If one writes “the cat sat on the …”, different words have different probabilities of continuing the sentence. In this instance, “mat” would be a far more likely continuation of the sentence compared to “neutrino”. This is obvious to us, but the surprising thing is that an artificial system trained using such a simple basic strategy (i.e., predicting what will come next) can emulate highly complex human reasoning. This is evidenced by their use in diverse fields such as chemistry [16], medicine [17, 18] and education [19].

In the present paper, we focus on the on the threat AI poses in the educational sector. There have been numerous anecdotal reports of academics running their assessment questions through AI systems such as GPT-4, grading the answers and finding that these systems attain excellent grades [20]. A recent rigorous blind study backed up these anecdotal reports by showing that ChatGPT produces comparable or better responses to exam questions than students across a range of subjects [21]. This study was experimentally based and did not test a real-world end-to-end examination system or measure the detectability of AI by educators. However, clearly, if students can simply copy and paste an assessment question into a system such as GPT-4 and in seconds get an output that will pass the assessment undetected, passing the assessment demonstrates absolutely no knowledge on the part of the student.

Academic assignments are typically classed into two broad categories: “coursework” and “exams”. Coursework assignments are traditionally completed with little to no supervision e.g., students are given an assignment question, go away to research the topic independently and write their answer over a period of weeks. Therefore, other than asking students not to use it via standard academic policy, there is nothing to stop students from using AI in completing their coursework. In contrast, up until very recently, exams were traditionally run “in person” under direct supervision, which precludes the use of AI without very elaborate use cases. This all changed in 2020 with the start of the COVID-19 pandemic; overnight, this required exams to move online and be completed at home.

In some ways, the COVID-19 pandemic simply accelerated an existing trend in the educational sector to organise exams online branding these as “authentic forms of assessment”, “constructively aligned” with real-life professional experiences [22]. Assessments should require students to demonstrate logical reasoning, integration of information, critical analysis, and the application of their knowledge to real world concerns. In person exams have been designed to reflect this, however, the take home online exam format reduces performance being partially a measure rote memorisation. The format is also more inclusive to those, for example, with mental health conditions or external responsibilities that make it difficult to attend an exam on campus (although alternative arrangements for students such as these have been in place prior to the move to online exams).

In addition to the potential educational benefits, in a complex business landscape, some universities have continued to run exams online with questions largely akin to those that students would get in a traditional supervised exam for non-pedagogical reasons as well. These include “enhancing the student experience” and “hearing the student voice”—student reported “experience” and “voice” being central to many metrics universities are judged upon [23, 24]. Such as, in the UK, the National Student Survey. This benefits universities that are competing for student tuition fees in an increasing overstretched and underfunded sector, described in the UK by the House of Lords as facing a “looming crisis” [25]. Furthermore, with increasing student numbers, there are increasing costs and practical efficiencies in not having to organise in person exams, which put pressure on university infrastructure.

Whatever the motivations, one cannot escape the fact that if AI can produce answers to assessments in a matter of seconds, students could easily complete unsupervised assessments using AI, whether these are classed as exams or coursework. Today’s AI can already demonstrate attributes such as integration of information and critical analysis [21], which are the very attributes educators want students to demonstrate. AI is also “multimodal” being able to analyse images and text and reason on the information these contain. It can integrate information from multiple resources, offer reasoning to why it came up with the answer it provided (and why it rejected others), and provide multimodal answers in response. In this context, AI renders unsupervised assessments, branded “authentic” [22] or not, dangerously susceptible to academic misconduct.

Academic misconduct has always been a problem. For example, students submitting someone else’s work as their own, colluding with one another, sharing questions, or purchasing answers from an “essay mill”. However, LLMs and AI pose a fundamentally different threat. They are widely available, (typically) free to use, trained on a huge corpus of data way beyond what peers or an essay mill could provide and can be used by students to interactively and iteratively complete assessments. LLMs also learn from the experience of the prompts submitted so can constantly improve (leading to companies such as Samsung to ban employees from using AI chatbots due to the accidental leakage of internal trade secrets [26]). Post-COVID with both exams and coursework being unsupervised, the unique threat of AI has led many institutions to move exams back to being in person to help maintain their academic integrity.

### Can the use of AI be detected?

If assessments and exams are primarily completed unsupervised, without the use of some kind of remote proctoring software and process, the question becomes whether there is a way to detect the use of AI in student submissions? If the use of AI can easily be detected, in some senses there is no real problem–students cheat using AI and then get caught. Whilst LLM’s are impressive, they are not perfect. They are reliant on the corpus of data they are trained on and do not logically plan their answers. Rather, in answering a query, they essentially predict what words (or more specifically “tokens”) will come next based upon what they have learned from their training data (for a detailed description see [27]). As a result, they can state ‘facts’ and ‘reasoning’ in response to a query, but the ‘facts’ can be made up and the ‘reasoning’ completely illogical. This type of error has been described as the AI “hallucinating” in its answers. Indeed, OpenAI state on the ChatGPT interface that “ChatGPT may produce inaccurate information about people, places, or facts”.

Several competing AI systems have been released with their creators claiming that these systems can successfully detect the use of AI in the production of written text. One of these was produced by OpenAI, the creators of GPT-4, but quietly withdrawn, due to its “low rate of accuracy” [28]. Given a piece of AI-written text the detection AI would correctly classify it as produced by AI only 26% of the time. This is in some senses unsurprising as the aim of LLMs is to mimic the production of human language. Turnitin, a global company that provides software for online assessment submission and grading have released an AI detection tool, initially claiming that it could detect “97 percent of ChatGPT and GPT-3 authored writing, with a very low less than 1/100 false positive rate” [29]. However, after releasing the detection software in beta they found that “real-world use is yielding different results from our lab” and that “in cases where we detect less than 20% of AI writing in a document, there is a higher incidence of false positives” [30].

There is also evidence that AI detection tools can exhibit bias against non-native English writers, classifying them more frequently as AI compared to native English writers [31]. Additionally, AI systems exist that are designed to make AI produced written text undetectable to the AI systems designed to detect AI. Recent research [32] has shown that that the latest AI detectors cannot reliably detect LLM outputs in practice, stating that “for a sufficiently advanced language model, even the best detector can only perform marginally better than a random classifier”. If detection is impossible, or at best highly problematic, this is particularly concerning because the potential consequences of academic misconduct can be high, with a huge population of students. At the time of writing, another widely discussed company with the aim of detecting AI generated text, GPTZero, explicitly states that their AI detection systems “should not be used to punish students”, instead recommending that “… educators take approaches that give students the opportunity to demonstrate their understanding in a controlled environment and craft assignments that cannot be solved with AI”.

### The present study

In this paper we report a real-world ‘Turing Test’ of the integrity of a university examinations system to infiltration by an AI chatbot (GPT- 4). To do this, we injected 100% AI written submissions into our examinations system in five undergraduate modules, across all years of study for a BSc degree in Psychology in the School of Psychology and Clinical Language Sciences (henceforth, ‘the School’) at the University of Reading (henceforth, ‘the University’). Markers of the exams were completely unaware of this. Overall, we found that 94% of AI submissions were undetected, even though we used AI in the most detectable way possible. AI submissions consistently outperformed real students, attaining grades which were on average around half a classification boundary higher. We found that in 83.4% of instances the grades achieved by AI submissions were higher than a random selection of the same number of student submissions.

## Methods

### Ethics

After reviewing a full written protocol, the University Ethics Committee Lead deemed this a quality assurance exercise not needing research ethics submission. Therefore, to gain authorisation to run the study we consulted the University Pro Vice Chancellor for Education, our Teaching and Learning Deans, the co-chairs of the University Sub-Committee on Delivery and Enhancement of Learning and Teaching, and our Head of School. We received unanimous approval to go ahead.

By design, markers on the modules we tested were completely unaware of the project. Other than those involved in authorising the study and the authors, only a handful of others were aware (e.g. those who helped arrange paid marking cover for the additional AI submissions and those who created the special university student accounts needed for AI submissions). Study authorisation did not require informed consent from markers. Following the analysis of the data, we invited all markers to two sessions chaired by our Head of School, to explain the study and gather feedback. Markers were very supportive and engaged in fruitful discussions. None were aware of the study having been run.

### Submitted exams

We were limited to submitting AI written answers to online exams where the additional marking load could be offset by bringing in additional paid markers. We submitted questions to all such exams where this was possible (Table 1). The proportion of AI written answers was approximately 5% of the total for each module. This percentage was decided upon (1) as it was a feasible number of submissions for two of the authors (Scarfe and Roesch) to submit live in each exam window (see below), and (2) marking load for this number of submissions could be offset. We were also wary of submitting large numbers of AI written answers which were self-similar due to not being modified (see below).

**Table 1:** Shows summary information for the five modules to which AI produced answers were submitted. For each module the “P” number is the part number (year of study) of the exam and the “M” number an index of the module e.g., P1-M1 is the module number 1 at part 1 that we submitted AI written answers to.

Module	Exam Type	Number of real submissions	Number of AI submissions	Percentage AI
**P1-M1**	SAQ	295	16	5.1
**P1-M2**	SAQ	294	16	4.8
**P2-M1**	SAQ	253	14	5.1
**P2-M2**	Essay	250	14	5.6
**P3-M1**	Essay	42	3	7.1
**Total**	---	1134	63	---

There were two classes of exam submitted to (1) Short Answer Questions (SAQs), where submission consisted for four answers from a choice of six questions, each with a 200-word limit and (2) Essay Based Questions, where submission consisted of a single 1500-word essay (students submitted one answer out of a choice of either three or four (dependent on the module)). SAQ’s were completed in a 2.5hr time limited window starting at 9am or 2pm. Essay exams were completed over an 8hr time limited window starting at 9am. Both were at home exams where students had access to their course materials, academic papers, books, the internet and could potentially collude and collaborate with peers or use generative AI.

### Procedure

We used a standardised prompts to GPT-4 to produce answers for each type of exam. For SAQ exams the prompt was:

*Including references to academic literature but not a separate reference section*, *answer the following question in 160 words*: *XXX*

For essay-based answers the prompt was:

*Including references to academic literature but not a separate reference section*, *write a 2000 word essay answering the following question*: *XXX*

In each prompt, XXX was replaced by the exam question. Note that the requested word limits in the prompts do not match the word limits in Table 1. This is because we found that with the correct word limit specified, for SAQ answers GPT-4 tended to produce too many words and for essay answers too few. With 160 words for the SAQ prompt GPT-4 tended to produce answers approximately on the target word count.

For essay answers, we were unable to get chat GPT-4 to produce 1500 words reliably even with increasing the word count in the prompt substantially. We therefore settled on 2000 words to marginally increase the words produced and prompted GPT-4 with “Please continue your response” if the answer produced was substantially short compared to our testing. This was done until it reached an approximately correct wordcount. We then simply collated the two or three answers provided, without adding any transition or altering the text in any way.

One essay question for module P3-M1 required one AI submission to contain an image illustrating the application of Norman’s principles of interaction design [33]. For this submission, we selected a picture of a revolving door with push plates, and adapted the prompt accordingly:

*Including references to academic literature but not a separate reference section*, *write a 2000 word essay answering the following question*: *“Explain why you think a revolving door with push plates is well-designed according to Norman’s principles of design*.*”*

The original exam question being:

*Find ONE object you think has been well-designed according to Norman’s principles of user-centred design*. *Provide a picture of the object and explain why you think it is well-designed according to those principles*.

Unlike SAQs, which had to be copy-pasted on an online submission form, essays had to be submitted as a separate document. We therefore applied minimal formatting, by changing the typeset of section titles, and using Microsoft Word’s build-in styles a different way for each submission.

For both types of exam students were encouraged to, where possible, include in-text citations, but a reference section at the end of the answer is not required. Occasionally, despite the tailored prompt, GPT-4 would produce reference sections at the end of the answers. When this happened, these were removed. Other than this, answers produced by GPT-4 were submitted unmodified given limited means to mimic student editing. We were also interested in the detectability of 100% AI written answers and the grades these submissions would receive, as this is a baseline for the infiltration potential of AI. None of the AI generated responses included the specific disclaimers that ChatGPT-4 sometimes adds, e.g., specifying that as an AI cannot do certain things.

For submission, AI alias university student accounts were set up for 33 students by our Digital Technology Services team. AI aliases were assigned to different year groups to ensure that they did not submit to incompatible modules. To ensure uniform coverage, we used a sliding window to determine which questions each fake student submitted on each module e.g., for a 6-question SAQ where 4 must be answered, student 1 answered 1–4, student 2 answered 2–5 with looping. Where more than one AI alias submitted an answer to the same question, we used the “regenerate” button of GPT-4 to produce multiple answers. No generated answers were discarded.

AI generated answers were submitted at the end of the submission period, on the day of each exam, using the special university student accounts. Exams were run on two standard commonly used submission and marking systems used across universities internationally. One system was used for SAQ exams and one for essay exams. The system used for essay exams had a similarity metric which gives a percentage value as to how much each submission matches a large database of external sources. The use of these systems for each type of exam was not related to any aspect of this study, but rather, standard procedure for how different exams were organised. Markers were, as standard, academic staff and paid trained PhD students [see also 21]. All marking was completely anonymous. Regardless of exam type, to the markers, our AI submissions procedurally looked identical to any other student submission.

### How were exams graded?

Examinations were graded on the University’s standard stepped numerical scale (Table 2). Submissions were graded by a first marker who read the work and assigned a grade according to how well the student had met the objectives of the assessment in line with the marking rubric. Grades were then moderated by an independent moderator who considered the overall level of attainment across submissions, read a randomly selected proportion of the submissions, and reviewed submissions that had attained a failing grade or had been flagged by the first marker (e.g., flagged for potential academic misconduct). The role of the moderator was not to double mark the submissions, but to review the quality, consistency, and appropriateness of the marking.

**Table 2:** This table shows the stepped grading scale used to mark individual questions. Where an exam has multiple questions, the overall mark is calculated as a weighted average of the marks for individual questions. For all exams in the present study all questions were weighted equally when calculating the overall mark.

Letter Grade	Degree classification	Percentage
A*	First (1^st^)	100
A++		95
A+		85
A		75
A-		72
B+	Upper Second (2:1)	68
B		65
B-		62
C+	Lower Second (2:2)	58
C		55
C-		52
D+	Third	48
D		45
D-		42
E	Marginal Fail	38
F+	Clear Fail	32
F		20
F-		10

Prior to marking commencing on modules with multiple markers, a “calibration session” was held for the marking team. Calibration involves a small number of submissions being graded by the full marking team and the grades awarded compared across markers. Where different marks are awarded, markers work to reach consensus on the appropriate grade to award. This ensures that all markers are applying the marking criteria consistently. Moderation serves as a further check on the consistency of the different markers on each module.

### What were markers told about AI?

At the time of running the study, in the summer 2023, the use of AI to complete exams was not allowed and fell under the standard University academic misconduct policy, which stated that the work submitted by students had to be their own. The software systems used for exams submission and grading did not have an “AI detector” component. Colleagues received standard guidance from the School about how to spot poor academic practice and academic misconduct. This including, (1) checking if answers sounded “too good to be true” e.g., a writing style, level of content, or quality, not expected from an undergraduate student completing a timed exam paper, (2) spotting answers which covered predominantly content which was not taught on the module, and (3) citations to references not linking with the claims being made in the answer. Many of these are characteristics of AI written text.

At the time, AI (particularly ChatGPT) was in the news media daily and an active topic of conversation amongst colleagues doing exam marking. The problem posed by AI for the academic integrity of assessments had also been discussed in larger meetings in the School. In debrief sessions given to colleagues who had marked on modules where we submitted AI (after the study had finished), virtually all were aware of the threat of AI to the integrity of exams. Indeed, in the few times where academic misconduct was suspected and reported, some colleagues referred to suspicions related to AI e.g., answers that seemed too “good to be true”, cited esoteric literature not covered in the course or cited seemingly non-existent references. Some had also run exam questions through ChatGPT to compare with the suspicious answers and/or run suspicious answers through online “AI detectors”. Note that this was not used in a diagnostic fashion for academic misconduct.

Discussions with academic colleagues at other universities and feedback from our external examiners [34] suggest that knowledge of AI among our colleagues and the policies in place to support its detection at the University were highly reflective of those across the sector at the time the study was run. Staff at Higher Education institutions across the UK were aware of the threat posed by AI but were not prepared for its rapid emergence and evolution as a tool that might be used by students to cheat in assessments. Universities were yet to put plans in place to handle the threat to academic integrity associated with misuse of AI tools or to integrate the use of AI into the curriculum.

## Results

### Were AI submissions detectable?

**Fig 1:**
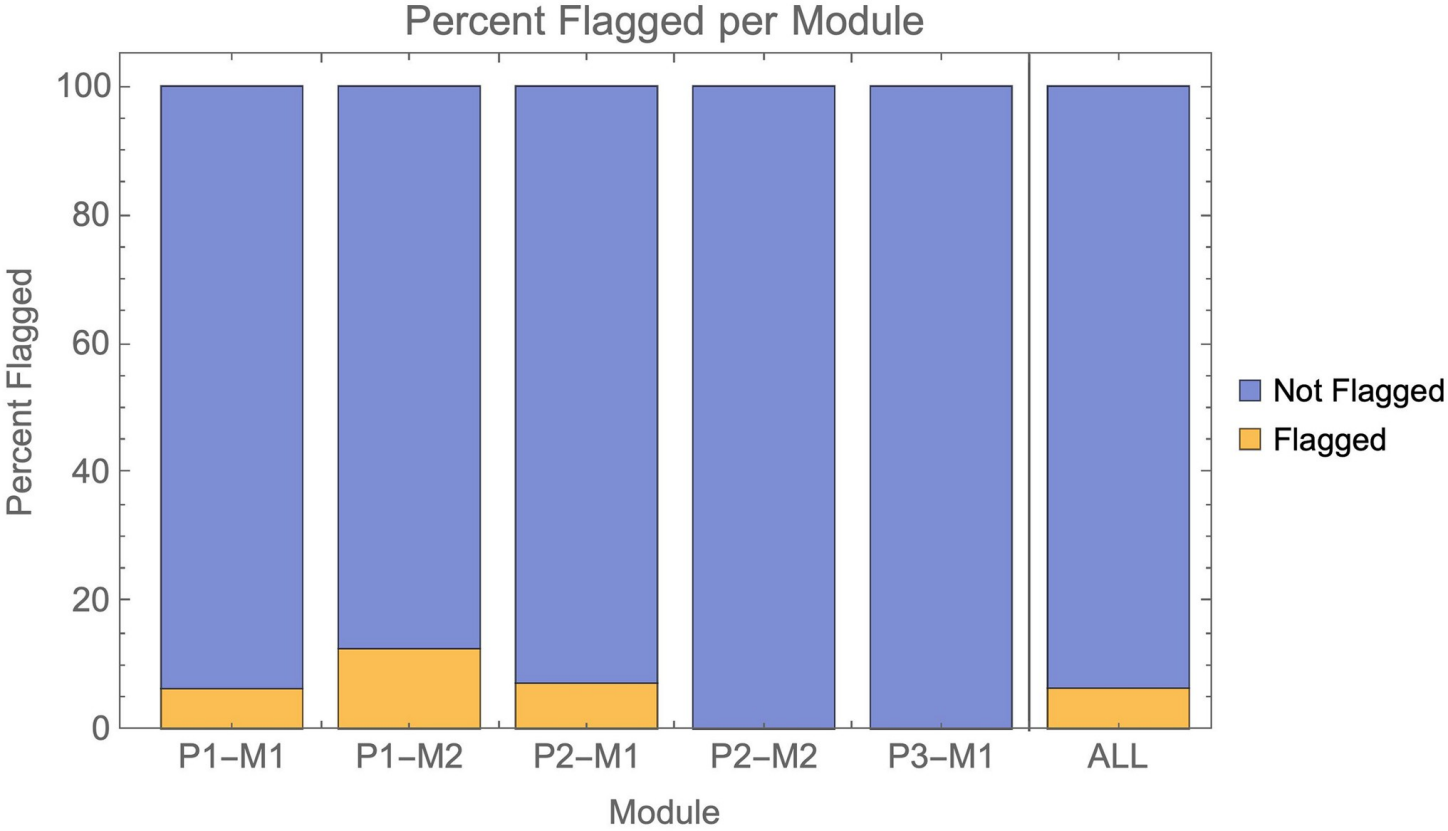
Shows the percentage of AI submissions flagged across individual modules, as well as overall. Flagging could be for any reason a marker had concerns about the submission based upon the standard academic misconduct policy. Markers did not have to mention AI in the flagging.

For the purposes of this study, to be classed as “detected” AI submissions needed only to be flagged for some form of poor academic practice or academic misconduct via standard university procedures. The marker did not need to mention AI when they flagged the script as of concern. The percentage of AI submissions detected in each module is shown in Fig 1 and Table 3.

**Table 3:** Shows detection statistics for the five modules to which AI produced answers were submitted. As in Table 1, for each module the “P” number is the part (year of study) of the exam and the “M” number an index of the module.

Module	Number flagged	Percentage flagged	Number flagged and AI mentioned	Percentage flagged and AI mentioned
**P1-M1**	1	6.25	1	6.25
**P1-M2**	2	12.5	0	0
**P2-M1**	1	7.14	1	7.14
**P2-M2**	0	0	0	0
**P3-M1**	0	0	0	0
**Overall**	**4**	**6.35**	**2**	**3.17**

**Fig 2:**
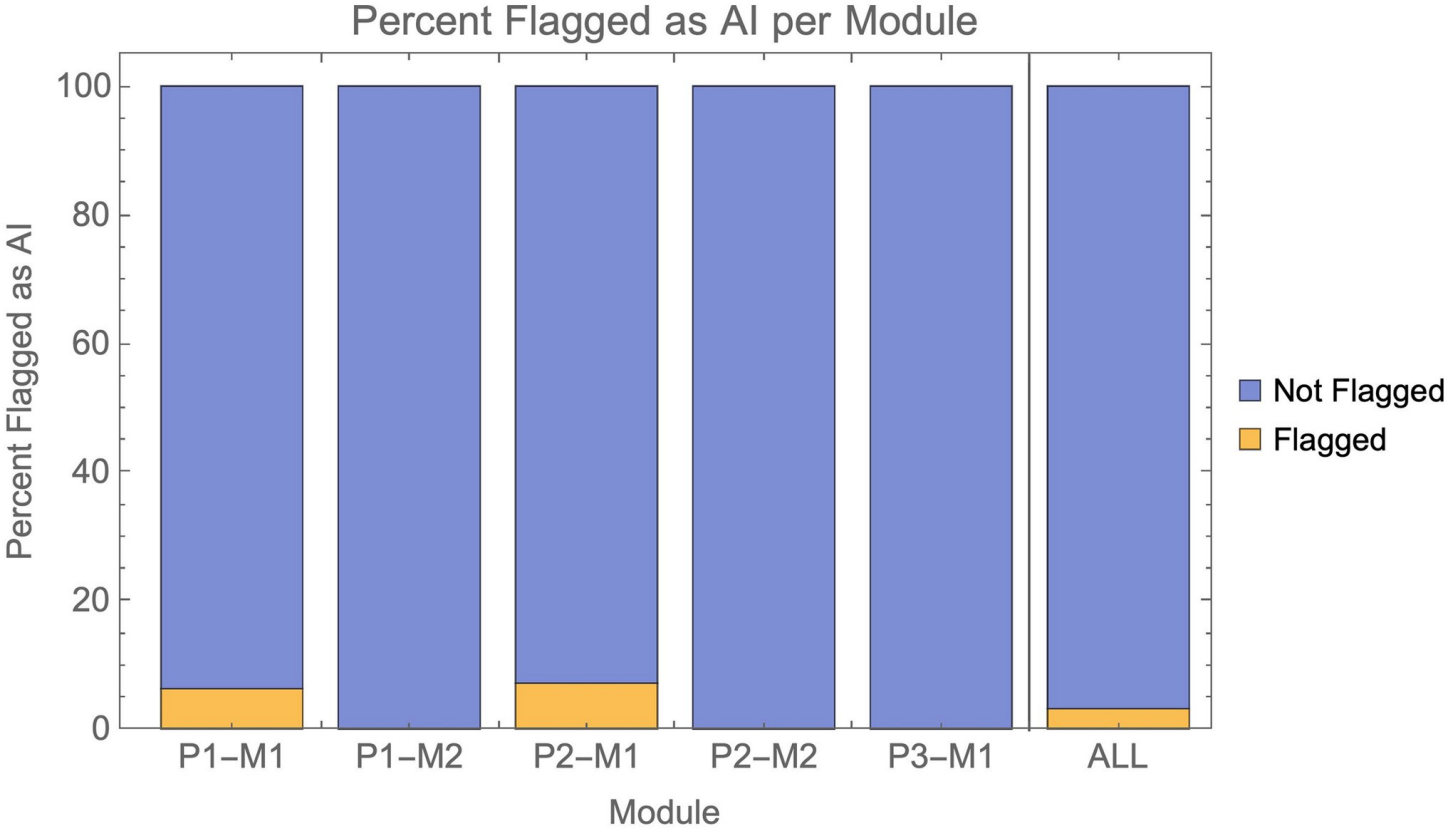
Shows the percentage of AI submissions flagged with any mention of AI across individual modules, as well as overall.

Overall, AI submissions verged on being undetectable, with 94% not being detected. If we adopt a stricter criterion for “detection” with a need for the flag to mention AI specifically, 97% of AI submissions were undetected (Fig 2).

### What grades did AI submissions attain?

**Fig 3:**
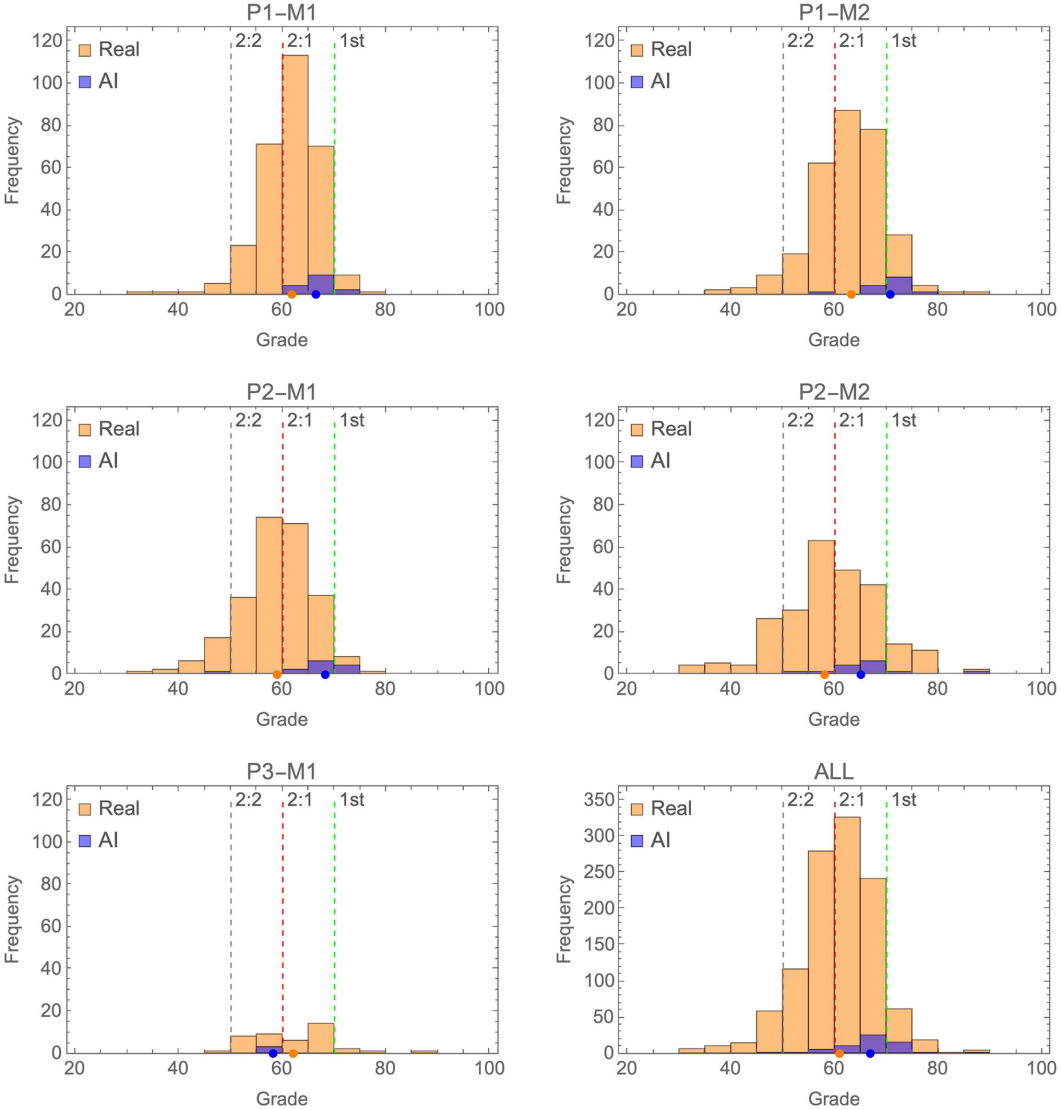
Shows grade frequency histograms for real (orange) and AI (blue) submissions across each individual module and all combined. Medians of the distributions are shown as dot in matching colours at the bottom of each plot. Grade boundaries for 2:2, 2:1 and 1^st^ classifications are shown as dotted lines.

Grade frequency histograms for each of the modules where AI was submitted are shown in Fig 3. Orange distributions are for real students and blue distributions fake AI students. The four detected AI submissions have been removed from all reported plots. As can be seen, AI submissions tended to attain grades at the higher end of the distribution of real students, gaining 2:1 to 1^st^ level grades. They tended to be clustered at this higher level rather than dispersed amongst the wider distribution of student grades. The exception to this was P3-M1 where AI submissions attained grades towards the lower end of the distribution for real students.

**Fig 4:**
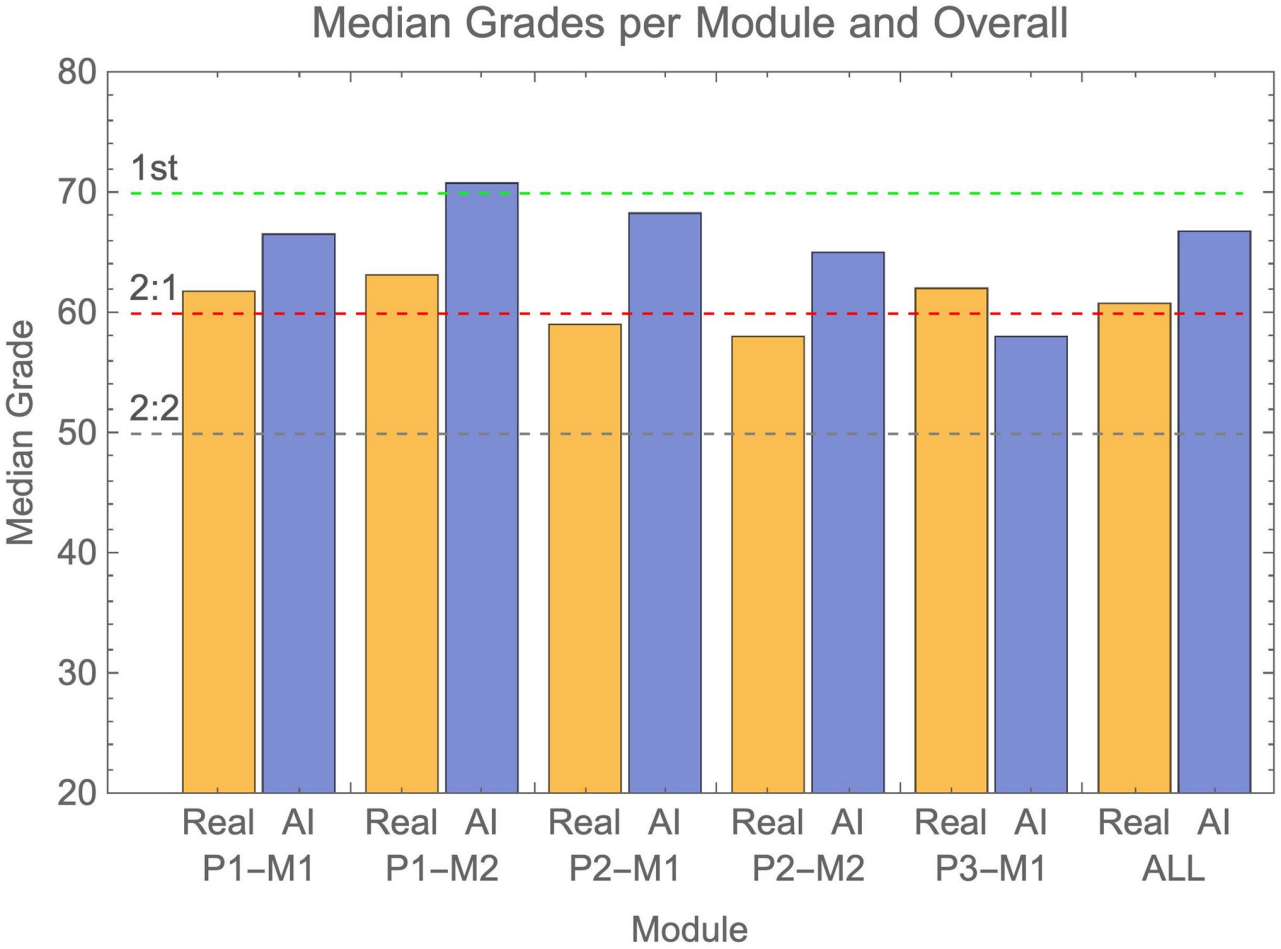
Shows median grades attained by real (orange) and AI (blue) submissions across each individual module and all combined. Grade boundaries for 2:2, 2:1 and 1^st^ classifications are shown as dotted lines.

Median grades attained by real and AI students are shown in Fig 4 (and as dots in Fig 3). In both plots grade boundaries for 1^st^, 2:1 and 2:2 level grades are shown as dashed lines. For four out of five modules AI submissions on average gained higher grades than real students, with medians in the 2:1 and 1st classification range. The exception being the finalist module P3-M1.

**Fig 5:**
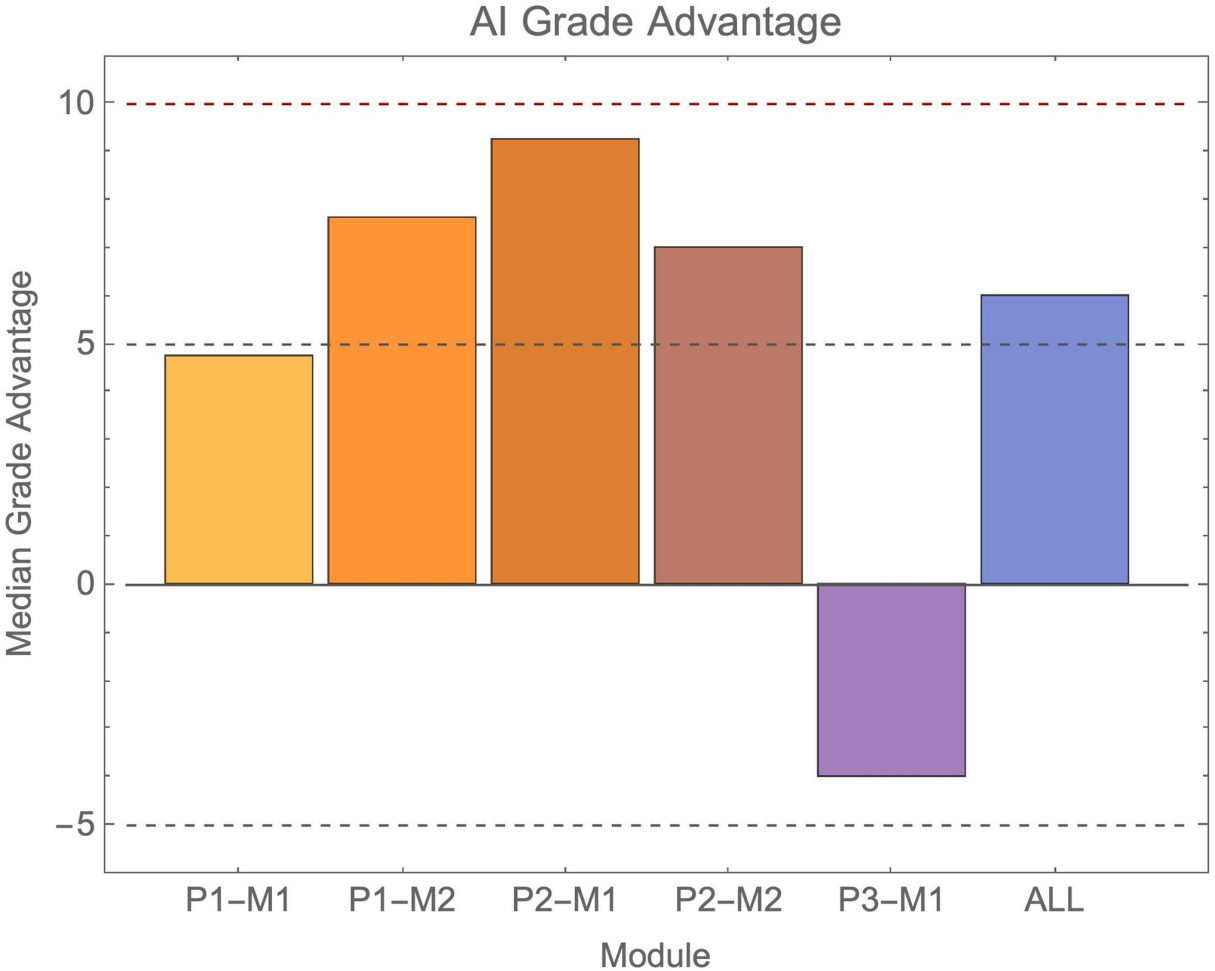
Shows relative grade advantage of AI submissions over real student submissions across all modules and overall. The dashed lines show a full and half classification boundary (10 and 5 percentage points respectively).

The magnitude of the advantage the AI submissions had over real students is shown in Fig 5. On average grades achieved by AI submissions were just over half a classification boundary higher than that achieved by real students, though this varied across modules (Fig 5). In the most extreme difference between AI and real students, the AI advantage approached that of a full grade boundary. This was in the module P1-M2 where the AI overall gained 1^st^ class grades.

### How likely was it that AI submissions attained a higher grade than real student submissions?

We can get an idea of the likelihood of AI outperforming real students through the following. For each module we have *n*_*m*_ AI submissions. We can therefore calculate the likelihood with which a random sample of *n*_*m*_ real student submissions from the module would be outperformed by the AI submissions on the same module. By “outperformed” we mean the median of the AI submissions being greater than the median of the selected student submissions. Thus, the process for each module is as follows: (1) randomly select *n*_*m*_ real student submissions, (2) calculate the median grade of this selection, (3) see if the median grade for the AI submissions on that module is greater than this, (4) repeat with replacement i.e., all real student submissions remain in the pool for selection on each repeat. Here we conducted this resampling process 100000 times.

**Fig 6:**
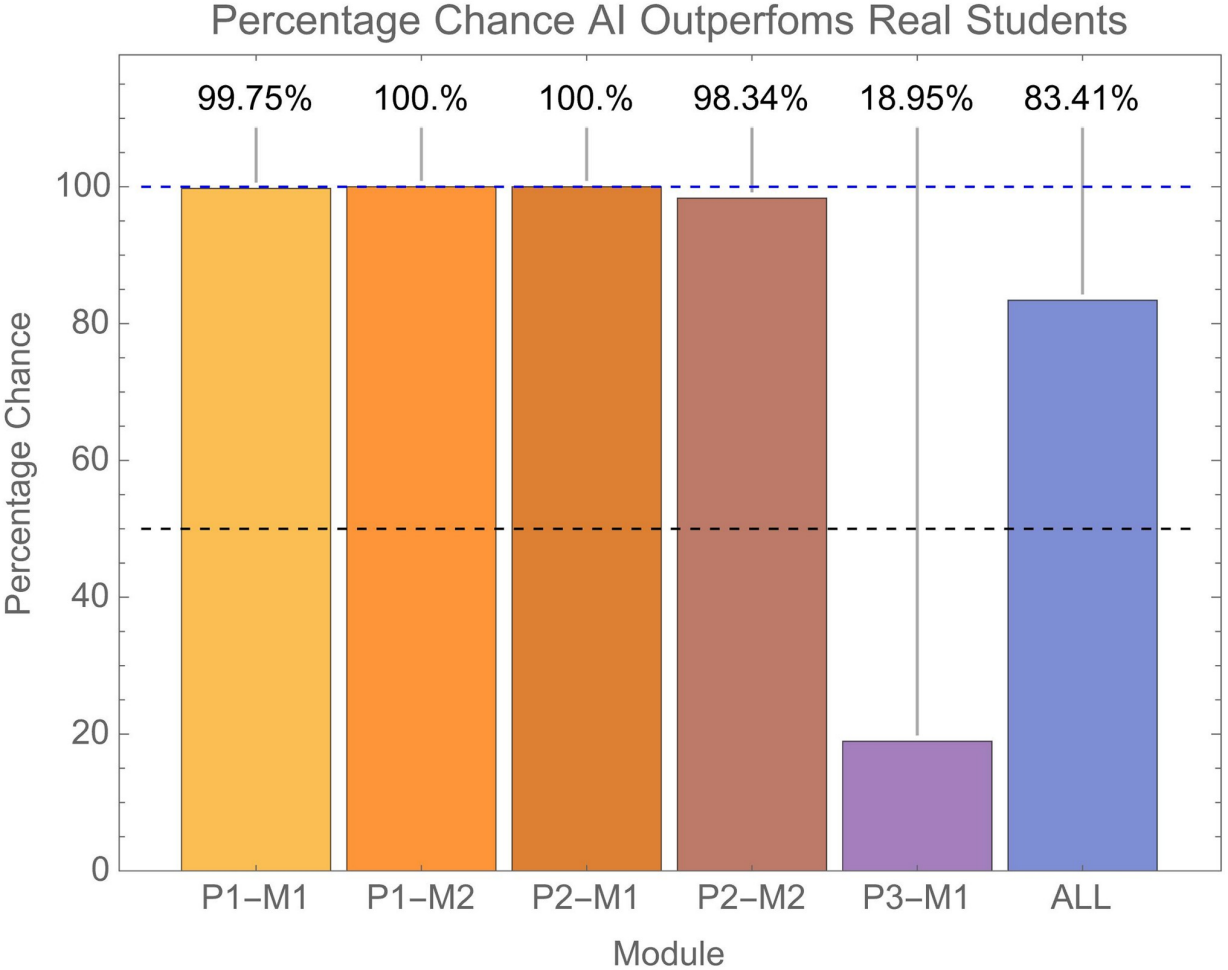
Shows the percentage chance that a random selection of *n*_*m*_ real student submissions would attain a lower median grade than the AI submissions. This is shown for individual modules as well as across all modules. The blue dashed line shows a 100% chance of AI outperforming students and the dashed black line a 50% chance.

The probability with which the AI submissions in each module would outperform a random selection of the same number of real student submissions is shown in Fig 6. For all modules bar P3-M1 there was nearly a 100% chance that a random selection of *n*_*m*_ real student submissions being outperformed by the *n*_*m*_ AI submissions. The exception was module P3-M1, where AI submissions outperformed real students in 19% of cases. Across modules as a whole, there was an 83.4% chance of a random selection of *n*_*m*_ real student submissions being outperformed by the *n*_*m*_ AI submissions.

**Fig 7:**
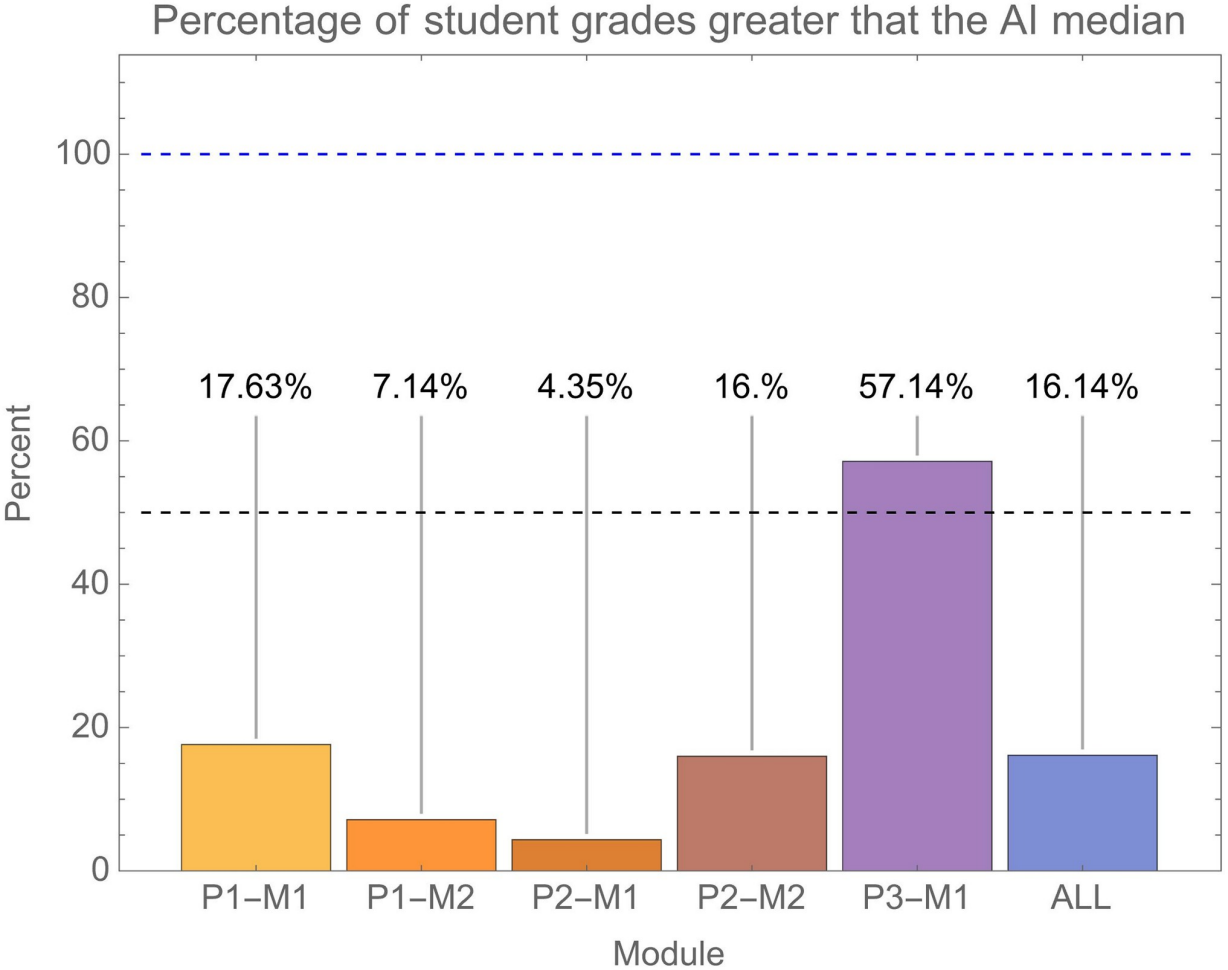
Shows the percentage of student grades greater than the median grade AI submissions attained. This is shown for each module and also overall. With reference to Fig 3, this is showing the percentage of student grades shown in the orange histograms which are greater than the median AI grade shown as the blue dot.

This result reflects the fact that AI submissions very consistently attained higher grades than real students. This is clear from grade histograms (Fig 3) where AI submissions tended to be clustered at the higher end of the distribution of grades attained by real student submissions. Indeed, we can simply calculate for each module (and overall) the percentage of student submissions that gained a grade greater than the median achieved by AI. This is shown in Fig 7. As would be expected, this produces results consistent with the resampling approach shown in Fig 6. Overall, only 16% of student submissions attained a higher grade than the median achieved by AI on the same module.

### Visualising grades and detectability

**Fig 8:**
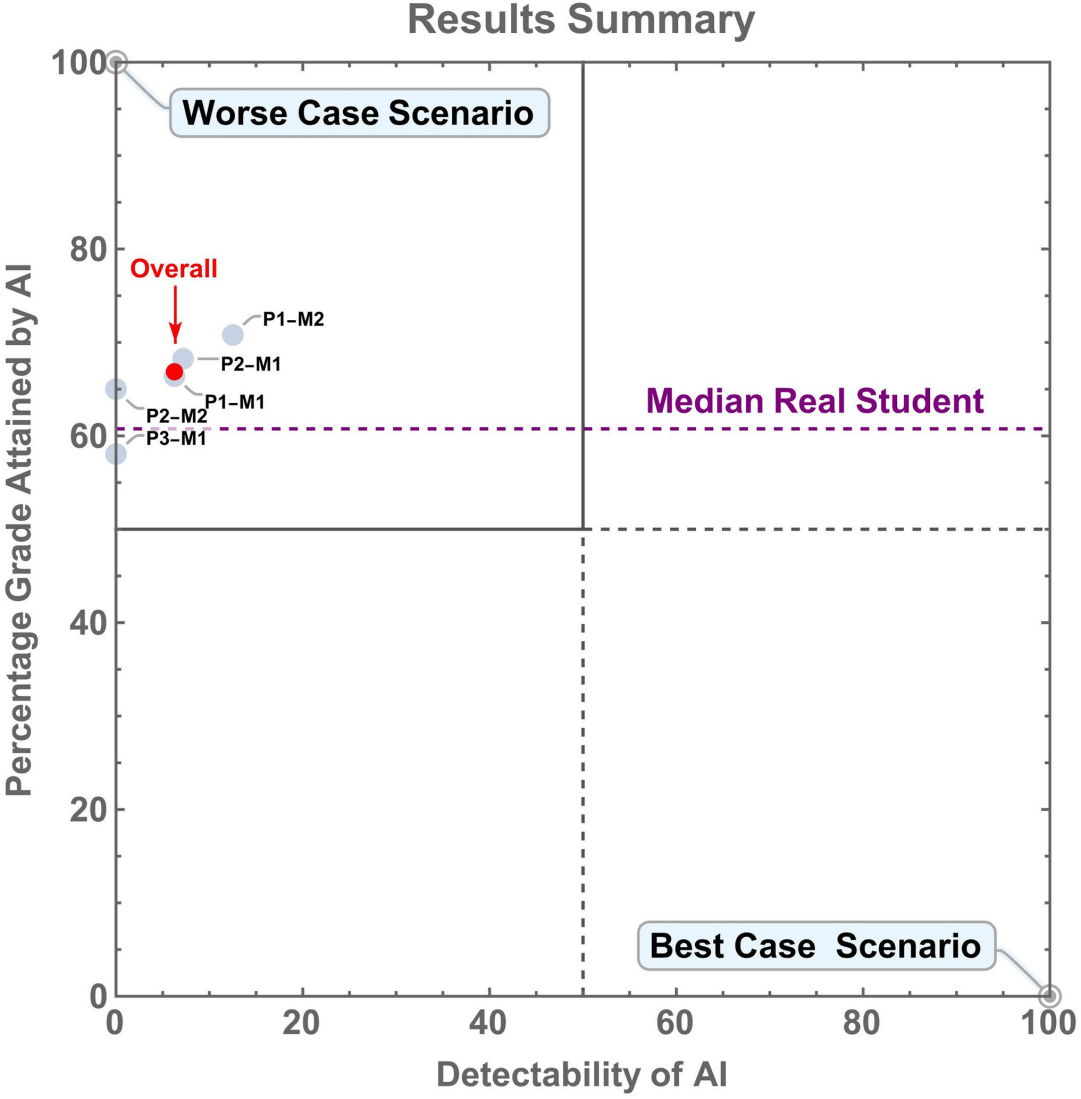
Summary of detectability for AI and the grades it achieved. The “best case scenario” is the lower right of the graph where AI is 100% detectable and gains 0% grades. The “worst case scenario” is the upper left of the graph where AI is undetectable and gains 100% grades. The data fall in the upper left quadrant of the graph (highlighted by solid lines) with AI being nearly undetectable and AI submissions consistently gaining higher grades than real student submissions (dashed purple line). This is shown for individual modules (labelled grey semi-opaque points) and overall (red point and arrow). The dashed horizontal purple shows the median grade attained by real student submissions over all modules.

Finally, to get an overall picture of both the grades attained by AI and its detectability, we can plot our data in a two-dimensional space (Fig 8). On the x-axis is the detectability of our AI submissions and on the y-axis the median grade that AI submissions attained. This is plotted for modules in isolation and overall. In terms of this space there is a clear order of preference for where we would like our data to be located. The “best case scenario” is that our data would be in the bottom right corner of the plot where AI is 100% detectable and attains 0% in terms of grade. An equally good situation for academic integrity perspective would be that our data were located at the top right corner of the plot. Here, students would cheat with AI and get a 100% grade, but we would detect their cheating 100% of the time.

A less ideal situation would be the bottom left corner of the plot, here we have no ability to detect the use of AI, but it attains a 0% grade. Therefore, students who cheated with AI would hopefully become incentivised not to, as they can attain higher grades by doing the work themselves. Finally, the “worst case scenario” is that the data fall in the top left corner of the plot. Here we have no ability to detect the use of AI and it attains a 100% grade. Clearly, we are in an area of this space where we do not want to be, with AI being nearly impossible to detect and consistently attaining higher grades than real students.

## Discussion

### Academic integrity

From a perspective of academic integrity, 100% AI written exam submissions being virtually undetectable is extremely concerning. Especially so as we left the content of the AI generated answers unmodified and simply used the “regenerate” button to produce multiple AI answers to the same question. A far more plausible strategy for academic misconduct would involve students modifying the AI output to make it less detectable, by, for instance, iteratively honing a series of prompts to construct an answer in collaboration with AI. If real students were cheating in an exam, they would be unlikely to take such a naively obvious approach as we did (unless they knew that they could, and we would not be able to detect it).

One might argue that no similarity check being available for SAQ exam submissions would impede marker’s ability to detect AI submissions. Our data provide no evidence for this. All AI submissions that were detected were detected in SAQ exams where no similarity check was available. None of the AI essay exam submissions were detected, where a similarity check was available. Overall, our 6% detection rate likely overestimates our ability to detect real-world use of AI to cheat in exams. This is particularly worrying as AI submissions robustly gained higher grades then real student submissions. Thus, students could cheat undetected using AI and in doing so attain a better grade then those who did not cheat.

The exception to the general pattern of AI outperforming students was our finalist module P3-M1. This is consistent with the notion that current AI struggles with more abstract reasoning [21], attributes at the higher levels of Blooms taxonomy [35, 36]. However, we are wary of speculating further based upon our data as this this was the only finalist module that we were logistically able to submit AI written answers to and it only had 3 AI submissions. This is clearly an interesting area for further study. Especially so in the rapidly improving landscape of LLM’s and AI more generally. Based upon current trends the ability of AI to exhibit more abstract reasoning is going to increase and its detectability decrease, meaning that the problem for academic integrity will get worse, necessitating action from educators now rather than later.

### What proportion of student’s cheat using AI?

Clearly, we have no way to estimate the proportion of students in our sample who used AI to complete their submissions. However, with the huge media coverage of AI, such as GPT-4, specifically in relation to cheating in exams, which included discussion of the failure of AI detectors, coupled with conversations with our students, we struggle to conclude that its use would be anything other than widespread. In a publicised freedom of information request, the University of Glasgow in the UK was found to have logged over 150000 connections to ChatGPT between May and the end of August 2023 [37]. This would be a substantial number of connections for students simply being curious about AI or doing independent research on AI themselves. Comparable data was not available from our university when requested.

Inferences can also be made based on student questionnaire reports as to the likelihood of them using AI. In a recent survey [21] it was found that in response to the question “Considering your next term of studies, would you use ChatGPT to assist with your studies?”, 74% of students indicate that they would use ChatGPT. The main reason students selected was to “improve my skills” (~40%) and “save time” (~30%). Only around 12% selected “better grades”. We would predict that these figures will change as knowledge of AI outperforming students increases. Especially as the most frequently selected reasons for students not using ChatGPT were “don’t know how to use it” (~31%) and “no need for it” (also ~31%). Furthermore, in this study students, compared to educators, were more likely to endorse the use of ChatGPT and less likely to endorse its use being acknowledged.

Therefore, pragmatically, it seems very likely that our markers graded, and did not detect, answers that students had produced using AI, in addition to our 100% AI generated answers. To counter this claim, one could argue that our AI submissions consistently outperformed real students, so this might suggest that AI was not used widely, else we would not have found this. An alternative argument is that students used AI, but in modifying the AI generated answers themselves made them worse than if that had simply entered the question and directly used the output unmodified as we did. Those student submissions which outperformed AI could also have consisted of AI generated material which students evaluated and verified, correcting factually incorrect information, to improve the AI response. Whatever the true nature of the reality in terms of the prevalence of use of AI, it is clear AI poses a serious threat to academic integrity.

### Ways to tackle AI / Can we tackle AI?

A simple and easy way to tackle the issue of AI academic misconduct would be to move back to supervised and in person modes of examinations. Indeed, some universities have already made this move after the necessity imposed by the COVID pandemic abated. This would not solve the problem of AI being used for coursework, which is completed largely unsupervised. Post COVID, many universities have however maintained a large proportion of exams being unsupervised ‘take home’ exams. These have normally taken broadly the same form as an in-person exam e.g., an essay question, but marked with knowledge that students would have access to course materials and the internet. There are several motivations for this, such as “enhancing the student experience” [23], making exams more inclusive, “hearing the student voice” [24], as well as the efficiency savings related to this in an increasingly underfunded and overstretched sector.

Whilst there is a pedagogical trend in the sector towards more “authentic” forms of assessment [22]. For example, practically demonstrating a skill rather than simply writing an essay about it. The “authentic” versus “unauthentic” distinction is quite different from the “supervised” versus “unsupervised” distinction. There can be highly “authentic” assessments which might be examined authentically in a traditional “pen and paper” setting. Equally, there are many “authentic” assessments which could be taken unsupervised and are trivial for AI to complete. Thus, tackling the problem of AI does not come down to pedagogical ideologies or categories. Rather, sound decisions should be *based upon evidence*.

Going forward AI will be used increasing in the workplace and it is highly likely, based upon current research, that we will be unable to detect students using AI to complete assessments. Framing AI as an addressable problem therefore seems misguided. A “new normal” integrating AI appears inevitable. An “authentic form of assessment” will be one in which AI is used. The question then becomes, how we can embrace the use of AI to enhance education rather than escape it? AI can have many benefits, for example, efficiently summarising large corpuses of data which it would take a human decades to read. This potentially frees up time for thinking more deeply about the material summarised by AI (so long as you can trust the summary). However, assessments will constantly need to be adjusted in the face of improvements in the extent to which AI can intelligently reason.

### “Trust” and “Truth” in an AI world

With the advent of a new technology, we must invariably take on responsibility for our ability to use that technology ethically and with clear knowledge of its limitations. We have described above how an AI such as GPT-4 can hallucinate facts and make seemingly logical arguments that are in fact completely illogical. It is limited by its training data. However, this is also true in how we as humans ingest information, we too have “a training data problem”. For example, if I based my understanding of the food preferences of the UK purely on the menu of McDonalds (one of the largest food chains in the UK), I would be missing a lot about the true state of the world. A common phrase in machine learning is “garbage in, garbage out”, illustrating that training on poor quality data typically doesn’t yield good outcomes.

Today’s AI has been made possible, in significant part, by training AI models on larger and larger corpuses of data. Pick a specialist topic and a human expert might do better than GPT-4, but in terms of overall “factual knowledge” GPT-4 “knows” vastly more than one human, or even a group of humans, could know in their lifetimes. AI currently makes mistakes, but so do we. The mistakes AI makes surprise us because they are “non-human” e.g., misclassifying an object in an image because a single pixel of the image has been changed [38], but GPT-4 would likely be surprised by our mistakes being very “non-AI”. In a world of “fake news” and “alternative facts”, we need to be cautious in simply passively taking what we read as fact, regardless of the source, AI or human.

## Conclusion

We have presented a rigorous real-world ‘Turing test’ for the infiltration of AI into the examinations system of a leading UK university. We found that within this system 100% AI written submissions were virtually undetectable and very consistently gained grades better than real student submissions. This poses serious questions for the educational sector if we are to maintain the academic integrity of our assessments. We have discussed our results in terms of our (or AI’s) ability (or not) to detect the use of AI and the need to acknowledge that in moving forward we likely need to accept the destination to be a “new normal” where the use of AI in generating the information we consume is inevitable.
